# Recent Advances in Statistical Theory and Applications

**DOI:** 10.3390/e25121661

**Published:** 2023-12-15

**Authors:** Augustine Wong, Xiaoping Shi

**Affiliations:** 1 Department of Mathematics and Statistics, York University, 4700 Keele Street, Toronto, ON M3J 1P3, Canada; 2 Department of Computer Science, Mathematics, Physics and Statistics, University of British Columbia, Kelowna, BC V1V 1V7, Canada; xiaoping.shi@ubc.ca

Complex data pose unique challenges for data processing. Moreover, traditional inferential methods need to be modified or extended in order to fully utilize information provided by the complex data so as to obtain accurate inference. The present Special Issue of *Entropy*, entitled *Recent Advances in Statistical Theory and Applications*, has captured some recent progress in clustering, change point inference, multiple sample tests, generalized linear model, and machine learning. All the papers included in this Special Issue are motivated by complex data that arise from stochastic processes, time series, and medical images.

Cluster analysis is a statistical method for processing data. The aim is to classify data into groups or clusters based on how closely associated they are. Measuring the closeness of data can vary, and it depends on many factors such as the researchers’ preferences and the type of data being analyzed. (contribution 1) considered the problem of obtaining an accurate clustering of unlabelled data. The common approach in solving this issue is ensemble clustering, which aims to combine sets of base clustering to obtain a better and more stable clustering and hence improve clustering accuracy. In this paper, the authors proposed a divergence-based locally weighted ensemble clustering with dictionary learning. Experimental results suggested that the proposed method is a promising method for ensemble clustering. (contribution 2) considered the problem of clustering mixed data that contain both continuous and discrete data, which arise frequently in medical and biological studies. It is an interesting topic of concern as most of the clustering methods found in the literature mainly target continuous data or discrete data alone. In this paper, using a weighted modified chi-squared test, the authors proposed a nonparametric clustering method to detect clustering patterns. Simulation studies showed that the proposed method outperforms AutoClass, which is the benchmark clustering method for mixed data. (contribution 3) examined data obtained from medical images, since an accurate and efficient clustering method will enable the early detection of possible tumors. It is well-known that the Euclidean distance-based Fuzzy C-means method is optimized to detect spherical structural clusters and does not perform well with high dimensional data. In this paper, the authors demonstrated that the Mahalanobis distance-based Fuzzy C-means method significantly outperformed the Euclidean distance-based Fuzzy C-means method.

A change point is a location or time at which observations obey two different models: before and after. Detecting change points is an interesting and important problem and has been studied by many authors. (contribution 4) incorporates prior information about the location of the change point to the current CUSUM-based statistics. The resulting class of weighted CUSUM statistics was shown to detect the change point with high accuracy. (contribution 5) combined neural network regression and location–scale CUSUM methods to detect the change point in autoregressive moving average models. Extensive simulations showed that the proposed method performs well.

Multiple sample tests exist frequently in statistical literature. The simplest example of a multiple sample test is the test of homogeneity of means using one-way ANOVA. However, when the underlying populations are not independently normally distributed, exact results may not be available. In relation to this, (contribution 6) assumed the populations are independently distributed as gamma distributions. The problem of the testing homogeneity of means is of great research interest, especially because gamma distribution is frequently used in survival data and engineering statistics. Exact results, however, were not available in this study. The author proposed an improved log-likelihood ratio test instead, and simulation results showed an exceptional accuracy for the proposed method. (contribution 7) focused on the homogeneity test that evaluates whether two multivariate samples come from the same distribution. In this paper, the authors proposed an improved test statistic based on data depth, which could be Mahalanobis depth, spatial depth, projection depth, etc. The advantage of the data depth approach is that it is free of strong distributional assumptions; therefore, the depth can provide a ranking of multivariate data. The simulation results conducted demonstrate the superior performance of the proposed tests.

A stochastic process is a collection of random variables indexed by time. Therefore, it is often used to model data with a dependence structure. In (contribution 8), a general statistical framework is provided for using active information to quantify the amount of pre-specified external knowledge that an algorithm uses in order to reach a certain target. By iterating a Metropolis–Hastings type of the Markov chain, the authors were able to compute the algorithm’s active information under the equilibrium and non-equilibrium of the Markov chain, with or without stopping when the targeted set of fine-tuned states has been reached. (contribution 9) applied a temporal self-exciting point process model to the terror data. Moreover, by using a combination of simulation and the random forest method, an accurate prediction of the number of terror events was obtained.

Some types of time series data pose instability and long-term unpredictability, leading to challenges in accurate estimation and inference. (contribution 10) considered the mildly explosive autoregressive process, which can be used to test the explosive behavior of economic growth. In particular, the authors performed theoretical studies on the properties of this model with strong mixing errors. The results are applied to data from the NASDAQ composite index. The subject matter of (contribution 11) is the chaotic time series model, which has the characteristics of internal randomness, nonlinearity, and long-term unpredictability. The authors investigated phase space reconstruction, model training, and model selection in a chaotic time series. Numerical results show that the proposed model performs very well in multi-step predictions.

A generalized linear model is a flexible generalization of an ordinary linear regression model by allowing the linear model to be related to the response variable via a link function and by allowing the magnitude of the variance of each measurement to be a function of its predicted value. Hence, the response variable is no longer restricted to being normally distributed. The author of (contribution 12) studied the problem of bilinear regression and extended it to the problem of inductive matrix completion, where the response matrix contains missing data. The author applied the quasi-Bayesian method to address the problem of bilinear regression, and then adapted this approach to the problem of inductive matrix completion. An efficient gradient-based sampling algorithm designed to sample from the quasi-posterior distribution was ultimately developed in the paper. (contribution 13) derived the asymptotic normality of the subsampling M-estimator through Fisher information. Moreover, the asymptotic properties of subsampling estimators of an unbounded generalized linear model with non-natural links were also studied.

Lastly, (contribution 14) applied the machine learning method to improve the accuracy in predicting winter precipitation. The methodology was applied to winter precipitation data collected in Northern China from 1997 to 2018. The results show that the proposed method outperformed the benchmark method.

The collection of articles in this Special Issue of *Entropy* illustrated that challenges arise from analyzing complex data, and new methodologies are proposed to solve the associated problems. We hope that the articles included in this Special Issue will provide readers with beneficial information and helpful ideas for their own research, and, most importantly, that readers enjoy reading these articles.

